# Peptaibols: Diversity, bioactivity, and biosynthesis

**DOI:** 10.1016/j.engmic.2022.100026

**Published:** 2022-06-05

**Authors:** Xuewen Hou, Ruonan Sun, Yanyan Feng, Runfang Zhang, Tianjiao Zhu, Qian Che, Guojian Zhang, Dehai Li

**Affiliations:** a Key Laboratory of Marine Drugs, Chinese Ministry of Education, School of Medicine and Pharmacy, Ocean University of China, Qingdao 266003, China; b Laboratory for Marine Drugs and Bioproducts, Pilot National Laboratory for Marine Science and Technology, Qingdao 266237, China; c Marine Biomedical Research Institute of Qingdao, Qingdao 266101, China

**Keywords:** Peptaibols, Structural diversity, Bioactivity, anti-infection, Biosynthesis

## Abstract

Peptaibols are a large family of linear, amphipathic polypeptides consisting of 5-20 amino acid residues generated from the fungal nonribosomal peptide synthetase (NRPS) pathway. With a relatively high content of non-proteinogenic amino acids such as *α*-aminoisobutyrate (Aib) and isovaline (Iva) in the skeleton, peptaibols exhibit a wide range of biological activities, including anti-microbial, cytotoxic, and neuroleptic effects. With five peptaibols brought to market for use as biocontrol agents, this class of peptides has received increasing attention from both biochemists and pharmacologists. In this review, we summarized the progress made in structural characterization, elucidation of biosynthetic pathways, and investigation of biosynthesis elucidation and bioactivities, to promote further efforts to develop peptaibols as pharmaceuticals.

## Graphical abstract

**Figure:**
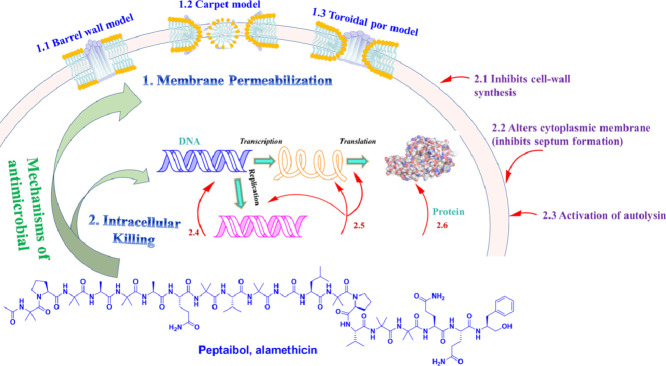

## 1 Introduction

Peptaibols are an intriguing class of fungal metabolites produced through the nonribosomal peptide synthetase (NRPS) pathway [90]. Peptaibols are the largest subgroup of peptaibiotics, which carry a C-terminal residue with a 2-amino alcohol [[26], [27],^30^]. The term “peptaibol” is constructed from several words that describe their structural characteristics: “peptide”, “*α*-aminoisobutyrate (aib)”, and “amino alcohol” [34]. With molecular weights ranging from 500 to 2200 Da, peptaibols usually consist of five to 20 amino acids and have relatively high levels of non-proteinogenic amino acids, such as Aib and Iva. In addition, peptaibols often have N-acyl and C-amino alcohol termini [24,115].

Peptaibols usually form special *α*-helix and *β*-bend patterns in their three-dimensional structures [109], which endow them with permeabilizing abilities. A range of biological functions are associated with these permeabilizing abilities, including antibacterial, antifungal, and neuroleptic properties, which facilitate substrate transport [4,44,101,103]. Peptaibols have received increasing attention from both biochemists and pharmacologists due to their wide range of bioactivities and the structural variability that can be generated by exchange of variable amino acid building blocks [29]. We here summarized the structural diversity, biological activities, and biogenetic studies that have been conducted on peptaibols, to gain a comprehensive understanding and support the ongoing research related to discovery of new peptaibols and development of peptaibols as pharmaceuticals.

## 2 Classification and structural determination of peptaibols

### 2.1 Classification of peptaibols

There are currently two main systems for classifying peptaibols. One widely used system is the immature classification method, which is based on sequences length; peptaibols are classified as “long”, “short”, and “lipo” peptaibol [86]. “Long” peptaibols are those containing 18−20 residues [78,84], whereas “short” peptaibols usually consist of 11−16 amino acids [52,108]. In some cases, peptaibols are also classified by the modification types on the terminal groups [24,115]. For example, peptaibols with N-termini acylated by octanoic, decanoic, or cis-dec-4-enoic acid are defined as lipopeptaibols. This group of peptaibols has been well summarized elsewhere (Fujita et al., 1994; Toniolo et al., 2001) and will therefore not be detailed here.

**Fig. 1:**
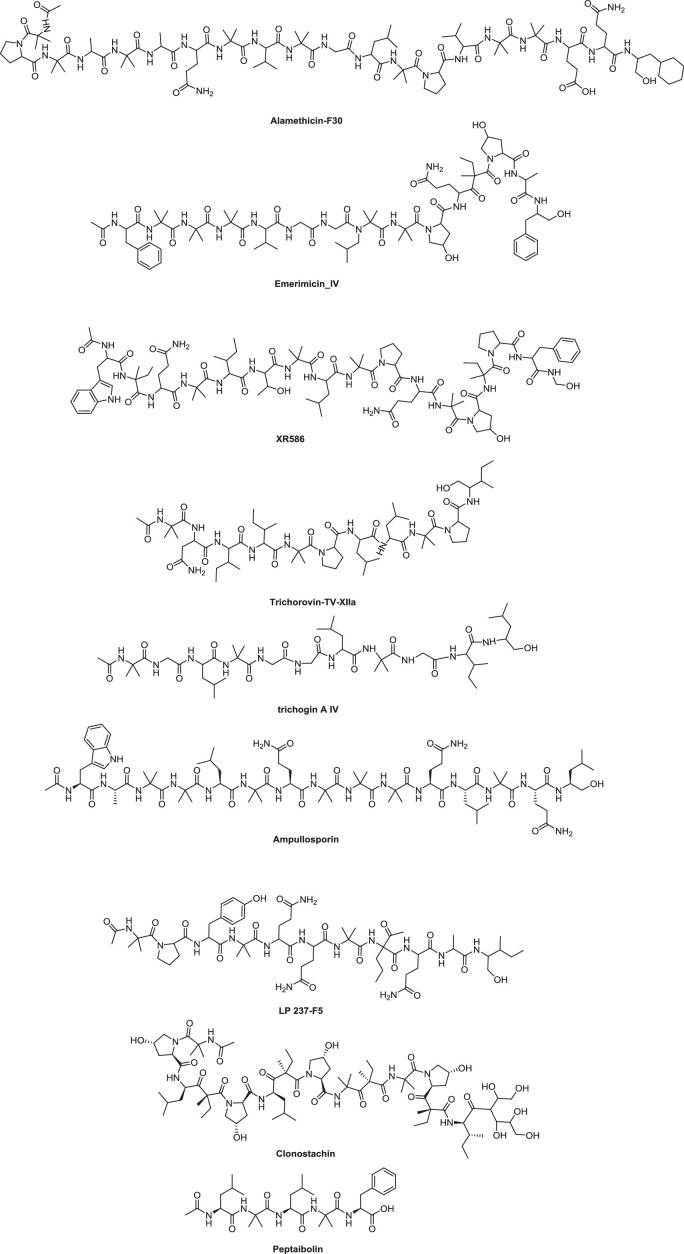
Structures of representative peptaibols in subfamilies (SFs) 1–9.

Classifications based only on sequence length do not encompass structures with 17 amino acids, such as cephaibol P [95], and rarely include information on structural features. To overcome this disadvantage, another classification method based on the similarity of residue and sequence length was proposed by Chugh and Wallace [22]. In this classification system, peptaibols are divided into nine subfamilies (SFs). This is convenient and efficient in systematic studies of the structures and components of peptaibols, such as the physical and chemical properties, biological activities and mechanisms. Representative sequences for SF1−9 and structures of peptaibol-based drugs (with their classifications) are summarized in Table 1 and Fig. 1**.** It should be mentioned that the Chugh and Wallace method was developed with the 200 peptaibols known in 2001, but there are more than 1000 known today. In the absence of a more accurate reclassification method, the Chugh and Wallace method remains a practical classification tool.

**Table 1:** Representative sequences of peptaibols in subfamilies (SFs) 1–9.

Subfamily (SF.)	Species	Peptaibols	Sequence	Refs.
SF1	*Trichoderma arundinaceum*	alamethicin-F30	Ac-*Aib*-Pro-*Aib-*Ala-*Aib*-Ala-**Gln**-*Aib*-Val-*Aib*-Gly-Leu-*Aib*-Pro-Val-*Aib*-*Aib*-**Glu-Gln**-Phe-OH	[82]
SF2	*Emericellopsis microspora*	emerimicin_IV	Ac-**Phe**-*Aib*-*Aib-Aib*-Val-**Gly**-Leu-*Aib-Aib*-Hyp-**Gln**-Iva-Hyp-Ala-**Phe**-OH	[88]
SF3	*Acremonium persicinum*	XR586	Ac-Trp-Iva-Gln*-Aib*-Ile-**Thr**-*Aib*-Leu-*Aib*-**Pro**-Gln-*Aib*-**Hyp**-Iva-**Pro**-Phe-Gly-OH	[97]
SF4	*Trichoderma arundinaceum*	trichorovin_TV_XIIa	Ac-*Aib*-**Asn**-Ile-Ile-*Aib*-**Pro**-Leu-Leu-*Aib*-**Pro**-Ile-OH	[115]
SF5	*Trichoderma longibrachiatum*	trichogin_A IV	Ac-*Aib*-**Gly**-Leu-*Aib*-**Gly**-**Gly**- Leu-*Aib*-**Gly**-Ile-Leuol	[110]
SF6	*Sepedonium ampullosporum*	ampullosporin	Ac-Trp-Ala-*Aib*-*Aib*-Leu-*Aib*-Gln-*Aib*-*Aib-Aib*-Gln-Leu-*Aib*-**Gln-Leu**-OH	[89]
SF7	*Tolypocladium geodesic*	LP 237-F5	Ac-*Aib*-**Pro**-Tyr-*Aib*-**Gln-Gln**-*Aib*-**EtNor**-**Gln**-Ala-Leu-OH	[124]
SF8	*Clonostachys* sp.	clonostachin	Ac-*Aib*-**Hyp**-Leu-Iva-**Hyp**-Leu-Iva-**Hyp**-*Aib*-Iva-*Aib*-**Hyp**-Iva-Ile-Mannitol	[20]
SF9	*Sepedonium spp*	peptaibolin	Ac-Leu-*Aib*-Leu-*Aib*-Phe-OH	[45]

Note: Amino acid residues in bold indicate the characteristic residue of each SF.

SF1 is the subfamily of “long” peptaibols, containing peptide sequences ranging from 17 to 20 residues. Members of this SF often carry Gln residues near the middle of sequences (position 6 or 7) and Gln−Gln or Glu−Gln pairs in positions 18 and 19. The presence of Gln or Glu is considered to be responsible for longer lifetimes of membrane channels [116]. Aib residues occur with high frequency in most SF1 sequences, resulting in predominantly *α*-helical configurations of the molecules. Aromatic amino acids are frequently seen in the C-termini or N-termini of some sequences, facilitating the formation of membrane channels [117].

The peptaibols in SF2 contain 14−16 residues and are also classified as “long” peptaibols, even though they are shorter than those in SF1. Similar to SF1, they have a high frequency of Aibs in the middle, and aromatic residues at both the N- and C-termini. The distinguishing feature of SF2 is the presence of conserved imino acids (Pro or Hyp) in positions 10 and 13.

Similar to SF2, members of SF3 also tend to have imino acids (in positions 10, 13, and 15) and aromatic residues at the N- and C-termini. However, one obvious difference between SF3 and SF2 is that SF3 usually has a conserved Thr residue in position 6, and in most cases, no Gly residue is present in the sequence [97]. SF4 consists of peptaibols with either 11 or 14 residues. This subfamily has a conserved Gln or Asn residue in position 2 and conserved Pro residues around positions 5 and 9.

SF5 consists of either 7 or 11 residues. This subfamily contains no charged residues such as Pro or Gln, but is Gly rich (Toniolo et al.). These Gly residues may line the lumen of a newly formed channel, permitting ions to bind with the polypeptide backbone instead of with side chains, thus affecting the permeabilizing ability.

Compared with SFs 1−5, SFs 6−9 are relatively small families, with fewer 10 members each. All SF6 sequences are 15 residues long. They have a higher frequency of Aib residues, compared with other peptaibols, and conserved Gln and Leu residues are usually found at positions 14 and 15 [10,63]. SF7 members contain an unusual amino acid and relatively high frequencies of Gln and ethyl-norvaline, which cause difficulties in aligning members of SF7 with other peptaibol sequences. The family usually carries conserved Gln residues in positions 5, 6, and 9. Members of SF8 have 14 residues and are rich in Hyp; no charged residues or aromatics are present, making this a family of extremely hydrophobic molecules [20]. The shortest peptaibols, members of SF9, have only five residues and therefore lack the amphiphilic permeabilizing function and the ability to insert into a membrane (Huelsmann et al., 1999).

### 2.2 Structural determination

Peptaibols usually have a relatively high molecular weight and complicated composition of amino acids, meaning it is challenging to accurately determine the structures. With the development of instrumental analysis technologies, it has become easier to determine the structures using a combination of different spectroscopic tools and chemical derivatization methods. High resolution electrospray ionization mass spectroscopy (HRESIMS^n^) and 1D and 2D nuclear magnetic resonance (NMR) are typically used to identify the composition and sequence of amino acids. Marfey's method is usually employed to determine the absolute configuration of amino acids. In rare cases, the structures of some oligopeptides can be conditionally determined by single-crystal X-ray diffraction.

#### 2.2.1 HRESIMS^n^ spectroscopic methods

In 2007, Juliana F. de S. Daniel developed the application of mass spectrometry in characterizing peptaibol structures [24]. In a typical (electrospray ionization-tandem mass spectrometry) ESIMS/MS spectrum for a peptaibol, the characteristic mass differences of 71.0 and 85.1 Da are observed, indicating acyl fragmentation and therefore the presence of Ala and Aib residues [57]. A mass difference of 113.1 Da indicates the presence of Ile, Leu, or Hyp amino acid residues [106]. Cleavage of the labile Aib−Pro or Aib−Hyp bonds often produce intense ions, from which a series of fragments can be generated by MS^2^ [100]. Based on those fragments, a putative peptaibol sequence can be hypothesized. When residues cannot be clearly identified with MS^2^ experiments, MS^3^ experiments can be employed to break special ions, producing more detailed fragments for further analysis. For example, Alexander Otto et al. first used positive and negative ion MS^2^ spectra, followed by a positive ion MS^3^ spectrum, to elucidate the sequence of chilenopeptins A as Ac-Aib^1^-Ser^2^-Trp^3^-Aib^4^-Pro^5^-Lxx^6^-Aib^7^-Aib^8^-Gln^9^-Aib^10^-Aib^11^-Gln^12^-Aib^13^-Lxx^14^-Pheol^15^ [76]. They also used this technique to aid in identifying the structures of four 11-residue peptaibols, albupeptins A–D [75]. Vincent P. Sica et al. used higher-energy collisional dissociation (HCD) to obtain in-source fragments, providing high resolution for different species of amino acids [100]. HRESIMS^n^ spectroscopic methods are the most convenient tool to generate preliminary information about amino acid composition and residue order.

During the past two years, two state-of-the-art methods for rapid screening and sequencing of peptaibiotics have been introduced: peptaibiomics [[28], [57]] and intact-cell MALDI-TOF mass spectrometry (IC-MS) [26]. Such LC/MS-based peptaibiomics approaches are based on the generation of so-called in-source fragments by collision-induced decomposition mass spectrometry (CID-MS) to the skimmer region of an electrospray ionization (ESI) mass spectrometer. Peptaibiomics involves growth of fungi on suitable media, solid-phase extraction of all peptaibiotics, high-performance liquid chromatography (HPLC) profiling, complete or partial ES-MS/MS sequencing of peptaibiotics, then sequence matching using data bases. Peptaibiomics and “Intact cell MALDI-TOF-MSH (IC-MS)” have considerably increased the screening efficiency for peptaibiotics, increasing our understanding of their structural diversity.

#### 2.2.2 NMR spectroscopic methods

NMR spectroscopic techniques are the preferred options to elucidate the structures of peptaibols. Heteronuclear Single Quantum Coherence spectroscopy (^1^H-^15^N HSQC) and Heteronuclear Multiple Bond Correlation (^1^H-^15^N HMBC) are typically used to detect the key correlation from hydrogen to nitrogen in one amide bond, which helps to connect amino acid residues ambiguously [85,91]. Total Correlation Spectroscopy (TOCSY) spectrum is commonly used to determine long spin system in peptide molecules. Correlations are seen between distant protons when there are couplings among every intervening proton. This is extremely useful for identifying protons on amino acids; all protons on a given peptide ring will have a correlation with all other protons on the same ring, but not with protons on different rings (*e.g.* hypomurocin A1, pseudokonins KL III and KL VI) [[8], [81],^85^]. Similar to TOCSY, two-dimensional Homonuclear Hartmann-Hahn spectroscope (^1^H-^1^H HOHAHA) is an effective tool to establish long distance connectivity. When combined with ^1^H-^1^H COSY, the proton signals that do not belong to the same coupling system can be eliminated by selecting the specific protons to irradiate, making it much easier to establish ^1^H-^1^H connectivity in small- and medium-size peptides [6]. The solvents used are important in NMR experiments. Dimethyl sulfoxide (DMSO-*d*_6_) and methanol-*d*_3_ are often chosen as solvents for NMR. They allow the amidic protons to be clearly displaced, simplifying analysis of the NMR spectra. This has been demonstrated with trichodermides, chilenopeptins, pseudokonins KL III and VI, and alamethicins [75,76,85,118]. Even in different solvents, amidic proton signals are usually present at *δ*_H_ 7.00−9.00 and carbonyl carbon signals at *δ*_C_ 170.0−180.0. Generally, the sharp methyl singlet signal is displayed between *δ*_H_ 2.00−2.04, indicating that the N-termini of peptides are acetylated. Methyl singlets are observed at *δ*_H_ 1.30−1.70 [35] and quaternary carbon resonances at *δ*_C_ 56.0−61.0, which could be attributed to Aib. Additionally, rotating frame overhauser effect spectroscopy (ROESY) or nuclear overhauser effect spectroscopy (NOESY) spectra can assist in determining the absolute configuration [4,100].

#### 2.2.3 X-ray diffraction method

X-ray diffraction is undoubtedly the most straightforward and conclusive tool to determine the molecular structure of a compound. However, due to restrictions associated with their chemical structures and physical properties, only some peptaibols have been determined by X-ray diffraction. These include the 10-residue aspereline D [19], the 14-residue trichovirin I-4 A [39], the 15-residue molecules samarosporin I [38] and ampullosporin A [58], the 16-residue molecules antiamoebin I [104], cephaibols A−C [100], and zervamicin Z−L [51], and the 18-residue Trichotoxin_A50E [21].

The atomic structure of samarosporin I has been unambiguously resolved at 100 and 293 K; the crystal structure mainly adopted a right-handed 3_10_-helix and a minor fraction of α-helical conformation with 10 intramolecular hydrogen bonds detected [38]. Cephaibols A, B, and C were all shown to adopt a helical conformation with a sharp bend (∼55°) at the central hydroxyproline; the spatial structures of these three crystals have small differences but share similar unit cell dimensions [100]. Zervamicins Z−L, a membrane ion-channel peptides isolated from *Emericellopsis salmosynnemata*, displayed four different crystal forms in different solvents. However, they all bent at Hyp^10^ (from ∼ 30°) in the helix, and formed water channels in a similar fashion. More detailed analysis into the structure-activity relationship suggested that the Gln in position 11 may control the opening and closing of the channel due to the unusual folding form [51].

#### 2.2.4 Combinations of methods

The complicated structures of peptaibols usually generate varied and overlapping signals, which make it difficult to accurately determine the structure using only one method. In these cases, the structure is usually elucidated with a combination of methods.

As conventional spectroscopic tools, NMR, MS, and CD analyses are often used in combination to make peptaibol structural assignments. As an example, the linkage sequences of chilenopeptins A and B were initially determined by HRESIMS^n^, but the amino acid at the sixth and fourteenth positions could not be determined for Leu or Ile residues due to the limitations of MS in distinguishing between constitutional isomers. In this case, 2D NMR provided additional evidence to define the amino sequence [76]. CD has also been used in combination with NMR to investigate the 3D structure of peptaibol structures with higher molecular weights. For antiamoebin I (a 16-residue peptaibol) in methanol solution, NMR data indicated a left-handed helix for residues 2–7 followed by a right-handed C-terminal helix [104]. However, a recent combination of NMR data with CD spectroscopy showed that the conformation of antiamoebin at the N-terminal rapidly changed between left-handed and right-handed 3_10_-helices, with an equal population in each state [98].

Marfey's method is the most frequently used chemical derivatization method to determine the absolute configuration of structures to complement NMR and MS experiments [43,53,74]. Two reagents, L-FDAA and Ru(D4-Por*)CO, are often selected for derivatization of amino acids [36,87]. With NMR and MS results defining the planar structure and Marfey's analysis solving the stereochemistry of amino acids, an entire group of peptaibols such as trichodermides A−E, lipovelutibols A−D, septocylindrins A−B, or asperelines A−F can be characterized [100,103,107,118]. Applying Marfey's method together with CD experiments allowed for the configurational assignment of microbacterins A and B [87].

Compared with chemical derivatizations, total synthesis can provide full structural information, aiding in unambiguous structural characterization of natural products. Within this scope, solid phase chemistry revolutionized peptide synthesis, and the structural characterization of peptaibols has undoubtedly benefitted from these developments. In 2016, Alexander Otto et al. discovered two new linear 15-residue peptaibols (chilenopeptins A and B) from the Chilean *Sepedonium aff. chalcipori* KSH 883. Total synthesis was conducted for these two compounds with a solid-phase approach, which confirmed the absolute configuration of all chiral amino acids as *L* [76]. In another study by the same group searching for new peptaibols, four 11-residue peptaibols, albupeptins A–D, were isolated from the fungus *Gliocladium album*. Here, solid-phase synthesis was also employed in combination with NMR and ESI-HRMS^n^ techniques to elucidate the structures unambiguously [75].

## 3 Bioactivity of peptaibols

**Fig. 2:**
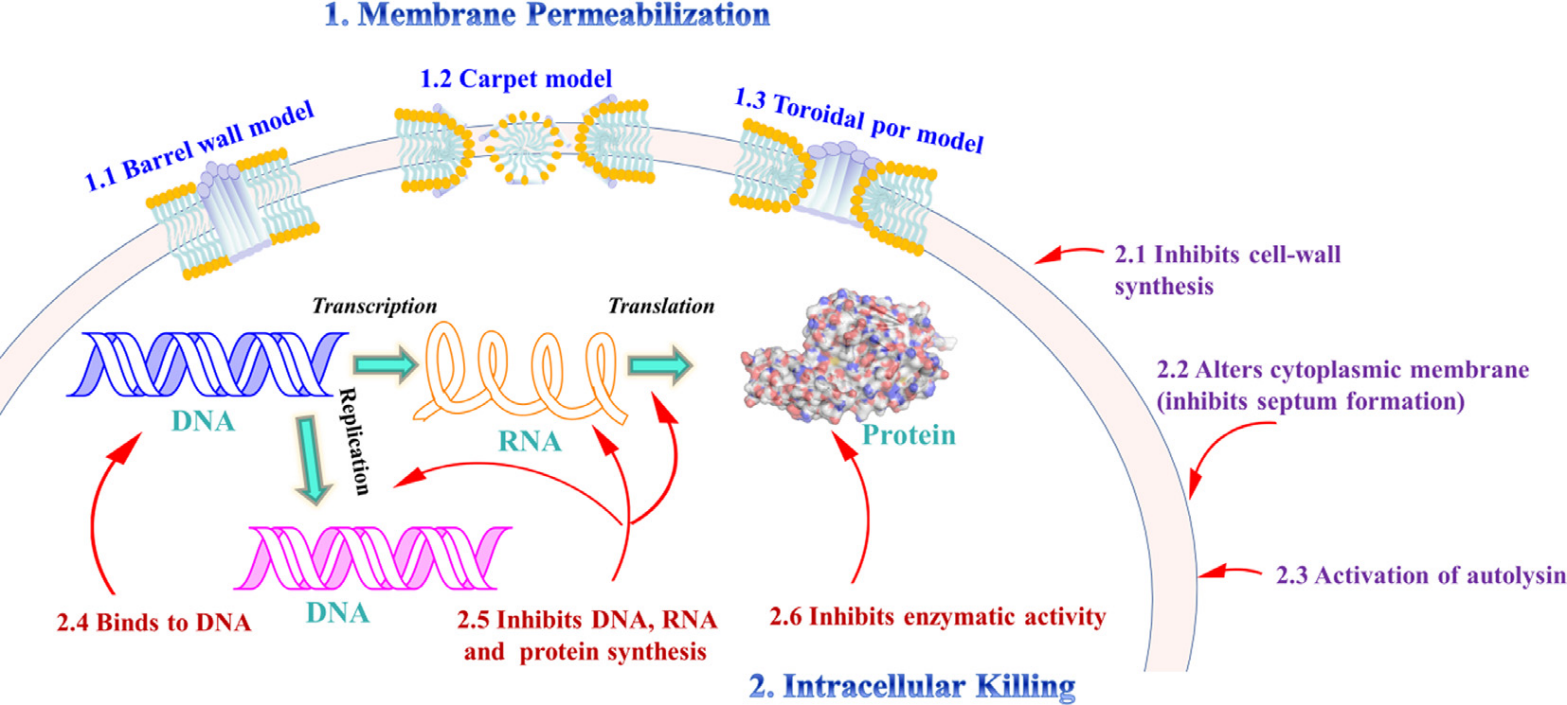
Membrane and non-membrane activity models of antimicrobial effects exhibited by peptaibols.

Peptaibols have received much attention from pharmacologists due to their various biological activities, which include antibacterial, antifungal, antitumor, and neuroleptic properties etc. However, based on published data from 1977 to 2021, most of the structures were assessed for their antimicrobial activity [[67], [101],^103^,^125^]. In this section, we review the pharmacological properties of peptaibols and the associated molecular mechanisms. Selected compounds with bioactivity and the associated modes of action are summarized in Table 2 and Fig. 2.

**Table 2:** Biological activity of peptaibols.

Compound	Biological activity (IC_50_ / MIC/ EC_50_)	Assay (organism/cell line)	Resource	Refs. /Years of discovery
Alamethicin F50	anthelmintic activity 0.2 *μ*g/mL (IC_50_)	L3 motility assay *Haemonchus contortus*	MSX 70741	[4]
Atroviridin B	anthelmintic activity 0.4 *μ*g/mL (IC_50_)	L3 motility assay *Haemonchus contortus*	MSX 70741	[4]
Longibranchin BIII	anthelmintic activity 3 *μ*g/mL (IC_50_)	L3 motility assay *Haemonchus contortus*	MSX 57715	[4]
Emerimicin IV	antimicrobial 12.5 *μ*g/mL (MIC)	microplate assay *E. faecalis*	*Emericellopsis minima*	[101]
	antimicrobial 100 *μ*g/mL (MIC)	microplate assay *S. aureus*	*Emericellopsis minima*	[101]
Trichokonins VI	antimicrobial 18.8 *μ*g/mL (EC_50_)	flat growth restraint assay *B. cinerea*	*Trichoderma pseudokoningii* SMF2	[125]
Chilenopeptins A	antimicrobial 5.3 ± 0.2 *μ*M(IC_50_)	microtiter plate assay *B. cinerea*	*Sepedonium* aff. *chalcipori* KSH 883	[76]
	antimicrobial 53.0 ± 4.1 *μ*M(IC_50_)	microtiter plate assay *S. tritici*	*Sepedonium* aff. *chalcipori* KSH 883	[76]
	antimicrobial 10.1 ± 0.3 *μ*M(IC_50_)	microtiter plate assay *P. infestans*	*Sepedonium* aff. *chalcipori* KSH 883	[76]
Chilenopeptins B	antimicrobial 7.0 ± 0.2 *μ*M(IC_50_)	microtiter plate assay *B. cinerea*	*Sepedonium* aff. *chalcipori* KSH 883	[76]
	antimicrobial 55.7 ± 6.2 *μ*M(IC_50_)	microtiter plate assay *S. tritici*	*Sepedonium* aff. *chalcipori* KSH 883	[76]
	antimicrobial 17.8 ± 1.0 *μ*M(IC_50_)	microtiter plate assay *P. infestans*	*Sepedonium* aff. *chalcipori* KSH 883	[76]
Septocylindrin A	antimicrobial 32 *µ*g/mL (MIC)	broth dilution method *Staphylococcus aureus*	*Septocylindrium* sp. LL-Z1518	[107]
	antimicrobial 16 *µ*g/mL (MIC)	broth dilution method *E. faecium* vancomycin-resistant	*Septocylindrium* sp. LL-Z1518	[107]
	antimicrobial 32 *µ*g/mL (MIC)	broth dilution method *E. coli imp* membrane permeability mutant	*Septocylindrium* sp. LL-Z1518	[107]
	antimicrobial 32 *µ*g/mL (MIC)	broth dilution method *Candida albicans*	*Septocylindrium* sp. LL-Z1518	[107]
Septocylindrin B	antimicrobial 8 *µ*g/mL (MIC)	broth dilution method *Staphylococcus aureus*	*Septocylindrium* sp. LL-Z1518	[107]
	antimicrobial 8 *µ*g/mL (MIC)	broth dilution method *E. faecium* vancomycin-resistant	*Septocylindrium* sp. LL-Z1518	[107]
	antimicrobial 8 *µ*g/mL (MIC)	broth dilution method *E. coli imp* membrane permeability mutant	*Septocylindrium* sp. LL-Z1518	[107]
	antimicrobial 16 *µ*g/mL (MIC)	broth dilution method *Candida albicans*	*Septocylindrium* sp. LL-Z1518	[107]
Trichokonins	antimicrobial	agar disk diffusion assay *B. subtilis*	*Trichoderma koningii* SMF2	[105]
Trichokonin VI	antimicrobial	agar disk diffusion assay *A. citrullinad*	*Trichoderma pseudokoningii* SMF2	[99]
	antimicrobial	agar disk diffusion assay *F. oxysporum*	*Trichoderma pseudokoningii* SMF2	[99]
	antimicrobial	agar disk diffusion assay *B. cinerea*	*Trichoderma pseudokoningii* SMF2	[99]
	antimicrobial	agar disk diffusion assay *P. parasitica*	*Trichoderma pseudokoningii* SMF2	[99]
	antimicrobial	agar disk diffusion assay *V. dahliae*	*Trichoderma pseudokoningii* SMF2	[99]
AcAib_8_CH_2_OTIPS **6**	antimicrobial	agar disk diffusion assay *B. megaterium*	Peptide synthesis	[2]
N_3_Aib_11_CH_2_OTIPS **11**	antimicrobial	agar disk diffusion assay *B. megaterium*	Peptide synthesis	[2]
N_3_Aib_12_C(O)OCH_2_CH_2_SiMe_3_**17**	antimicrobial	agar disk diffusion assay *B. megaterium*	Peptide synthesis	[2]
N_3_Aib_13_TMS **18**	antimicrobial	agar disk diffusion assay *B. megaterium*	Peptide synthesis	[2]
Hyporientalin A	antimicrobial 2.49∼4.92 *μ*M(MIC)	microplate assay *C. albicans* ATCC10231	*Trichoderma orientale* LSBA1	[48]
	antimicrobial 2.49∼4.92 *μ*M(MIC)	microplate assay *C. albicans* 247FN	*Trichoderma orientale* LSBA1	[48]
	antimicrobial 2.49∼4.92 *μ*M(MIC)	microplate assay *C. albicans* 098VC	*Trichoderma orientale* LSBA1	[48]
	antimicrobial 19.66 *μ*M(MIC)	microplate assay *C. albicans* 311FN	*Trichoderma orientale* LSBA1	[48]
Trichorzianine 1938	antimicrobial 12.5∼200 *μ*g/mL (MIC)	microplate assay *Sporosarcina* sp*.* (NB90)	*Trichoderma atroviride* NF16	[49]
	antimicrobial 12.5∼200 *μ*g/mL (MIC)	microplate assay *Bacillus* sp*.* (NB36)	*Trichoderma atroviride* NF16	[49]
	antimicrobial 12.5∼200 *μ*g/mL (MIC)	microplate assay *Axinella polypoides Microbacterium* sp. (PII.14)	*Trichoderma atroviride* NF16	[49]
	antimicrobial 12.5∼200 *μ*g/mL (MIC)	microplate assay Rhodobacteraceae (PI.03)	*Trichoderma atroviride* NF16	[49]
	antimicrobial 12.5∼200 *μ*g/mL (MIC)	microplate assay *Shewanella* sp. (PIII.07)	*Trichoderma atroviride* NF16	[49]
Trichorzianine 1909	antimicrobial 50∼200 *μ*g/mL (MIC)	microplate assay *B. subtilis*	*Trichoderma atroviride* NF16	[49]
	antimicrobial 12.5∼200 *μ*g/mL (MIC)	microplate assay *Sporosarcina* sp*.* (NB90)	*Trichoderma atroviride* NF16	[49]
	antimicrobial 12.5∼200 *μ*g/mL (MIC)	microplate assay *Bacillus* sp*.* (NB36)	*Trichoderma atroviride* NF16	[49]
	antimicrobial 12.5∼200 *μ*g/mL (MIC)	microplate assay *Axinella polypoides Microbacterium* sp. (PII.14)	*Trichoderma atroviride* NF16	[49]
	antimicrobial 12.5∼200 *μ*g/mL (MIC)	microplate assay Rhodobacteraceae (PI.03)	*Trichoderma atroviride* NF16	[49]
	antimicrobial 12.5∼200 *μ*g/mL (MIC)	microplate assay *Shewanella* sp. (PIII.07)	*Trichoderma atroviride* NF16	[49]
Trichorzianine 1895	antimicrobial 50∼200 *μ*g/mL (MIC)	microplate assay *B. subtilis*	*Trichoderma atroviride* NF16	[49]
	antimicrobial 12.5∼200 *μ*g/mL (MIC)	microplate assay *Sporosarcina* sp*.* (NB90)	*Trichoderma atroviride* NF16	[49]
	antimicrobial 12.5∼200 *μ*g/mL (MIC)	microplate assay *Bacillus* sp*.* (NB36)	*Trichoderma atroviride* NF16	[49]
	antimicrobial 12.5∼200 *μ*g/mL (MIC)	microplate assay *Axinella polypoides Microbacterium* sp. (PII.14)	*Trichoderma atroviride* NF16	[49]
	antimicrobial 12.5∼200 *μ*g/mL (MIC)	microplate assay Rhodobacteraceae (PI.03)	*Trichoderma atroviride* NF16	[49]
	antimicrobial 12.5∼200 *μ*g/mL(MIC)	microplate assay *Shewanella* sp. (PIII.07)	*Trichoderma atroviride* NF16	[49]
Trichorzianine 1896	antimicrobial 50∼200 *μ*g/mL(MIC)	microplate assay *S. albus*	*Trichoderma atroviride* NF16	[49]
	antimicrobial 50∼200 *μ*g/mL(MIC)	microplate assay *B. subtilis*	*Trichoderma atroviride* NF16	[49]
	antimicrobial 12.5∼200 *μ*g/mL(MIC)	microplate assay *Sporosarcina* sp*.* (NB90)	*Trichoderma atroviride* NF16	[49]
	antimicrobial 12.5∼200 *μ*g/mL(MIC)	microplate assay *Bacillus* sp*. (NB36)*	*Trichoderma atroviride* NF16	[49]
	antimicrobial 12.5∼200 *μ*g/mL(MIC)	microplate assay *Axinella polypoides Microbacterium* sp. (PII.14)	*Trichoderma atroviride* NF16	[49]
	antimicrobial 12.5∼200 *μ*g/mL(MIC)	microplate assay Rhodobacteraceae (PI.03)	*Trichoderma atroviride* NF16	[49]
	antimicrobial 12.5∼200 *μ*g/mL(MIC)	microplate assay *Shewanella* sp. (PIII.07)	*Trichoderma atroviride* NF16	[49]
Trichorzianine 1924	antimicrobial 12.5∼200 *μ*g/mL(MIC)	microplate assay *Sporosarcina* sp*.* (NB90)	*Trichoderma atroviride* NF16	[49]
	antimicrobial 12.5∼200 *μ*g/mL(MIC)	microplate assay *Bacillus* sp*.* (NB36)	*Trichoderma atroviride* NF16	[49]
	antimicrobial 12.5∼200 *μ*g/mL(MIC)	microplate assay *Axinella polypoides Microbacterium* sp. (PII.14)	*Trichoderma atroviride* NF16	[49]
Trichorzianine 1910	antimicrobial 12.5∼200 *μ*g/mL(MIC)	microplate assay *Sporosarcina* sp*.* (NB90)	*Trichoderma atroviride* NF16	[49]
	antimicrobial 12.5∼200 *μ*g/mL(MIC)	microplate assay *Bacillus* sp*.* (NB36)	*Trichoderma atroviride* NF16	[49]
	antimicrobial 12.5∼200 *μ*g/mL(MIC)	microplate assay *Axinella polypoides Microbacterium* sp. (PII.14)	*Trichoderma atroviride* NF16	[49]
	antimicrobial 12.5∼200 *μ*g/mL(MIC)	microplate assay Rhodobacteraceae (PI.03)	*Trichoderma atroviride* NF16	[49]
	antimicrobial 12.5∼200 *μ*g/mL(MIC)	microplate assay *Shewanella* sp. (PIII.07)	*Trichoderma atroviride* NF16	[49]
Trichorzianine 1924a	antimicrobial 50∼200 *μ*g/mL(MIC)	microplate assay *B. subtilis*	*Trichoderma atroviride* NF16	[49]
	antimicrobial 12.5∼200 *μ*g/mL(MIC)	microplate assay *Sporosarcina* sp*.* (NB90)	*Trichoderma atroviride* NF16	[49]
	antimicrobial 12.5∼200 *μ*g/mL(MIC)	microplate assay *Bacillus* sp*.* (NB36)	*Trichoderma atroviride* NF16	[49]
	antimicrobial 12.5∼200 *μ*g/mL(MIC)	microplate assay *Axinella polypoides Microbacterium* sp. (PII.14)	*Trichoderma atroviride* NF16	[49]
	antimicrobial 12.5∼200 *μ*g/mL(MIC)	microplate assay Rhodobacteraceae (PI.03)	*Trichoderma atroviride* NF16	[49]
	antimicrobial 12.5∼200 *μ*g/mL(MIC)	microplate assay *Shewanella* sp. (PIII.07)	*Trichoderma atroviride* NF16	[49]
Trichorzianine 1909a	antimicrobial 50∼200*μ*g /mL(MIC)	microplate assay *B. subtilis*	*Trichoderma atroviride* NF16	[49]
	antimicrobial 12.5∼200 *μ*g/mL(MIC)	microplate assay *Sporosarcina* sp*.* (NB90)	*Trichoderma atroviride* NF16	[49]
	antimicrobial 12.5∼200 *μ*g/mL(MIC)	microplate assay *Bacillus* sp*. (NB36)*	*Trichoderma atroviride* NF16	[49]
	antimicrobial 12.5∼200 *μ*g/mL(MIC)	microplate assay *Axinella polypoides Microbacterium* sp. (PII.14)	*Trichoderma atroviride* NF16	[49]
	antimicrobial 12.5∼200 *μ*g/mL(MIC)	microplate assay Rhodobacteraceae (PI.03)	*Trichoderma atroviride* NF16	[49]
	antimicrobial 12.5∼200 *μ*g/mL(MIC)	microplate assay *Shewanella* sp. (PIII.07)	*Trichoderma atroviride* NF16	[49]
Trichorzianine 1938	antimicrobial 12.5∼200 *μ*g/mL(MIC)	microplate assay *Sporosarcina* sp*.* (NB90)	*Trichoderma atroviride* NF16	[49]
	antimicrobial 12.5∼200 *μ*g/mL(MIC)	microplate assay *Bacillus* sp*.* (NB36)	*Trichoderma atroviride* NF16	[49]
	antimicrobial 12.5∼200 *μ*g/mL(MIC)	microplate assay *Axinella polypoides Microbacterium* sp. (PII.14)	*Trichoderma atroviride* NF16	[49]
	antimicrobial 12.5∼200 *μ*g/mL(MIC)	microplate assay Rhodobacteraceae (PI.03)	*Trichoderma atroviride* NF16	[49]
	antimicrobial 12.5∼200 *μ*g/mL(MIC)	microplate assay *Shewanella* sp. (PIII.07)	*Trichoderma atroviride* NF16	[49]
Trichokonins	antimicrobial	TMV infection tests Tobacco (Nicotiana tabacum var. Samsun NN)	*Trichoderma pseudokoningii* SMF2	[121]
Pentadecaibins I−V	antimicrobial 25∼100 *μ*g/mL(MIC)	microplate assay *S. aureus, E. coli, C. albicans*	*Trichoderma* sp.	[13]
Leucinostatin Z	antimicrobial	dual culture *B. cinerea*	*P. lilacinum*	[65]
Longibrachin-A-I	neurotoxicity ED_50¼_ 270 mg/kg	acute toxicity test the blue fly Calliphora vomitoria L.	*Trichoderma longibrachiatum* Rifai	[92]
Ampullosporin A	neuroleptic-like activity 2.5 mg/kg amp	conditioned avoidance response (CAR) Male Sprague–Dawley rats	*Sepedonium ampullosporum* HKI0053	[9]
Zervamicins IIA	neuroleptic activity 0.05 – 2.0 mg/kg	hole-board test male CD-1 mice	*Emericellopsis salmosynnemata* 336 IMI 58330	[77]
	neuroleptic activity 0.1 mg/kg	elevated Plus Maze (EPM) Test male CD-1 mice	*Emericellopsis salmosynnemata* 336 IMI 58330	[77]
	neuroleptic activity 0.1 mg/kg	forced-Swim Test male CD-1 mice	*Emericellopsis salmosynnemata* 336 IMI 58330	[77]
	neuroleptic activity 0.5 mg/kg	bar-Holding Test male CD-1 mice	*Emericellopsis salmosynnemata* 336 IMI 58330	[77]
Zervamicins IIB	neuroleptic activity 0.5 – 12.0 mg/kg	hole-board test male CD-1 mice	*Emericellopsis salmosynnemata* 336 IMI 58330	[77]
	neuroleptic activity 4.0 mg/kg	elevated Plus Maze (EPM) Test male CD-1 mice	*Emericellopsis salmosynnemata* 336 IMI 58330	[77]
	neuroleptic activity 4.0 mg/kg	forced-Swim Test male CD-1 mice	*Emericellopsis salmosynnemata* 336 IMI 58330	[77]
	neuroleptic activity 4.0 mg/kg	bar-Holding Test male CD-1 mice	*Emericellopsis salmosynnemata* 336 IMI 58330	[77]
Acrebol	mitochondrial respiratory chain inhibitory 80∼350 ng/mL(IC_50_)	MTT/ MIN-6	*Acremonium exuViarum* BMB4	[60]
Trichopolyn VI	mitochondrial respiratory chain inhibitory	paper disk assay *∆aac S. cerevisiae*	*trichoderma brevicompactum* FKI-6324	[106]
Peptaibols Alamethicin	facilitate substrate transport	phospholipid vesicular system test Substrate Bz-Arg-pNA	-	[59]
Zervamicin IIB	facilitate substrate transport	phospholipid vesicular system test substrate Bz-Arg-pNA	-	[59]
20-residue Trilongins	Na^+^/K^+^-permeable channels/0.4 *μ*g/mL(EC_50_)	boar spermatozoa motility inhibition test	*Trichoderma longibrachiatum*	[71]
11-residue Trilongins	Na^+^/K^+^-permeable channels 1.5 *μ*g/mL(EC_50_)	boar spermatozoa motility inhibition test	*Trichoderma longibrachiatum*	[71]
Velutibol A	antitumor 4-23 *μ*M(IC_50_)	MTT A549, LS-180, HL-60, MDA-MB-231	*Trichoderma velutinum*	[102]
Lipovelutibols B	antitumor 2 *μ*M(IC_50_)	MTT HL-60	*Trichoderma velutinum*	[103]
	antitumor 4 *μ*M(IC_50_)	MTT MDA-MD-231	*Trichoderma velutinum*	[103]
Lipovelutibols D	antitumor 4 *μ*M(IC_50_)	MTT HL-60	*Trichoderma velutinum*	[103]
	antitumor 7 *μ*M(IC_50_)	MTT LS180	*Trichoderma velutinum*	[103]
	antitumor 5 *μ*M(IC_50_)	MTT MDA-MD-231	*Trichoderma velutinum*	[103]
	antitumor 4 *μ*M(IC_50_)	MTT A549	*Trichoderma velutinum*	[103]
Trichokonin VI	antitumor/ 10∼20 *μ*M(IC_50_)	MTT HepG2	*Trichoderma pseudokoningii* SMF2	[70]
	antitumor/ 10∼20 *μ*M(IC_50_)	MTT BGC-823	*Trichoderma pseudokoningii* SMF2	[70]
	antitumor/ 10∼20 *μ*M(IC_50_)	MTT A549	*Trichoderma pseudokoningii* SMF2	[70]
Trichobrachin A-IX	antitumor/ 1.7 *μ*M(IC_50_)	MTT KB	*Trichoderma longibrachiatum* Rifai	[93]
Trichobrachin C	antitumor/ 0.8 *μ*M(IC_50_)	MTT KB	*Trichoderma longibrachiatum* Rifai	[93]
Culicinin D	antitumor/ <0.007 µg/mL (IC_50_)	MTS MDA468	*Culicinomyces cla Wisporus* LL-12I252	[40]
Acremopeptin	antitumor/ 0.78 *μ*M(GI_50_)	western blot method PC-3	*Acremonium* sp. PF1450	[47]
	antitumor/ 0.66 *μ*M(GI_50_)	western blot method HT-29	*Acremonium* sp. PF1450	[47]
Cyclodextrin-Scaffolded Alamethicin	membrane permeability	calcein release assay	peptide synthesis	[44]
Trichogin GA IV	membrane-perturbing activity	peptide-induced vesicle leakage experiments	peptide synthesis	[Bobone et a., 2013]

**Fig. 3:**
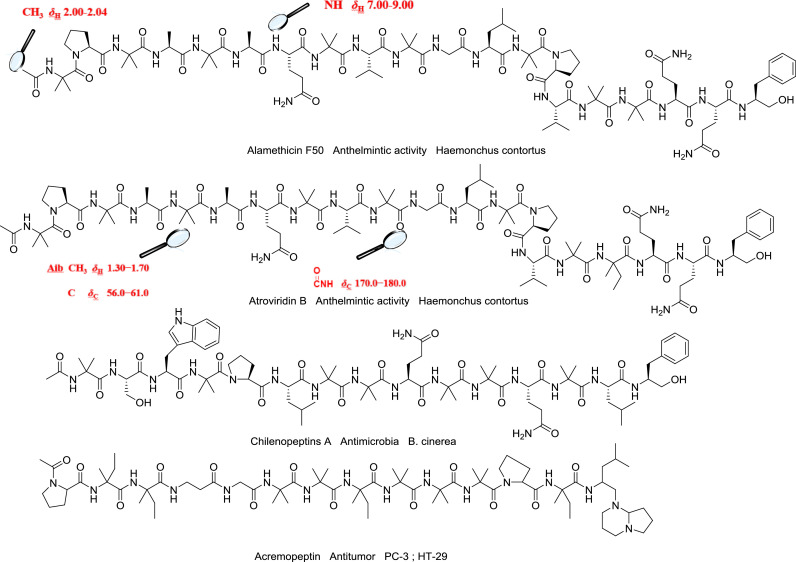
Selected peptaibols with intriguing activities and associated NMR characteristics.

### 3.1 Antibacterial and antifungal activity

In 2006, [105]. demonstrated that trichokonins exhibit antimicrobial activity against several fungal pathogens and discussed the mechanism of trichokonin VI. The result of mechanism indicated that it was a metacaspase-independent apoptotic to exhibit antimicrobial activity [99]. Previously, emerimicin IV was shown by Alejandro I. et al. to have antibacterial activity, especially against the multidrug-resistant (MDR) strains vancomycin-resistant *Enterococcus faecalis* (VRE) and methicillin-resistant *Staphylococcus aureus* (MRSA), against which the minimum inhibitory concentration (MIC) values were 12.5 μg/mL and 100 μg/mL, respectively. The mechanism of action was reported to be growth inhibition rather than bacterial killing [88,101].

Activity tracking method, separation by tracking active fractions, is a simple but sufficient method to separate peptaibols. Hyporientalin A, a peptaibol analogue of longibrachin-A-II, was separated by Ines Touati et al. from *Trichoderma orientale* based on this method; it displayed excellent activity against *Candida albicans*, and the MIC value was comparable to that of amphotericin B [48]. Summers et al. separated the novel septocylindrins A and B and the known peptaibol alamethicin, all of which exhibited significant antibacterial and antifungal activity [107]. Eight new peptaibols (trichorzianines) isolated from *Trichoderma atroviride* exhibited antimicrobial activity against Gram-positive bacteria [79]. Chilenoeptins A and B, the 14-residue peptides isolated from Chilean *Septonium* aff. *chalcipori* KSH 883, showed strong inhibitory activity against growth of the phytopathogenic organisms *Botrytis cinerea* and moderate inhibitory activity against *Phytophthora infestans* [76].

To study the relationship between antibacterial activity and structure, Adam et al. [2] designed different peptaibol-mimetic foldamers. Vesicle assays showed that long foldamers with hydrophobic termini containing Aib residues exhibited the highest ionophoric activity. N-terminal acetyl groups could allow Aib foldamers to form long-lasting and well-defined channels in planar bilayers and generally did not adversely affect ionophoric performance in vesicles or antibacterial behavior. In contrast, a C-terminal hydroxyl group mildly inhibited activity in the vesicle assays and significantly diminished antibacterial activity compared to C-terminal esters. Generally, peptaibols that included high rates of Aib residues and other non-proteinogenic amino acids such as Iva presented better activity.

### 3.2 Neuroactive properties

Ruiz et al. [92] reported that longibrachin-A-I could enhance the neurotoxicity of domoic acid when the concentration was under the minimal effective dose. The target for domoic acid and longibrachin-A-I was demonstrated to be ion channels [92]. Ampullosporin A was proven to have a neuroprotective effect [9]. It did not provoke the locomotor stimulation caused by the dopaminergic agonist apomorphine or suppress the hyperactivity caused by an NMDA receptor antagonist. Considering the amphipathic helix structure, the mechanism of action may be to form pores for potassium ions or interact with proteins. Ovchinnikova et al. [77] found that zervamicins can decrease spontaneous locomotor activity in mice; zervamicins consistently displayed neuroleptic activity in spite of dosage difference.

### 3.3 Antitumor activity

Trichokonin VI (TK VI), a peptaibol from *Trichoderma pseudokoningii* SMF2, inhibited growth of hepatocellular carcinoma cells (HCC) in a dose-dependent manner, but had little effect on normal liver cells at lower concentrations. This suggested that it may be a potential suppressor of tumor cells [70]. Lipovelutibols B and D, which contain a fatty acyl moiety at the N-terminus, were isolated from the Himalayan cold habitat fungus *Trichoderma velutinum*. They exhibited inhibitory activities against several tumor cells, such as HL-60, MDA-MD-231, A549, and LS180 [103]. Culicinin D, a 10-residue linear peptide isolated from the fungus *Culicinomyces clavisporus,* exhibited selective inhibitory activity against PTEN-negative MDA468 tumor cells [40].

Ruiz et al. [93] identified 30 11-residue peptaibols, among which trichobrachin A-IX and trichobrachin C exhibited the highest cytotoxic activity. Further research demonstrated an exponential relationship between, peptaibol hydrophobicity and cytotoxicity [93]. Through activity selection, Masatomi Iijima et al. isolated the 13-residue peptaibol acremopepin. Acremopepin suppressed the expression of survivin, an apoptosis inhibitor, and inhibited the growth of hormone-refractory prostate cancer PC-3 cells and colorectal adenocarcinoma HT-29 cells *in vitro* [47].

### 3.4 Functions in plants and animals

Trichokonins, isolated from *Trichoderma pseudokoningii* SMF2, can function as antimicrobial peptaibols and could induce defense responses and systemic resistance in tobacco against tobacco mosaic virus (TMV) infection. When tobacco was treated with trichokonins, the production of reactive oxygen species (ROS) and phenolic compounds increased. Additionally, Trichokonins enhanced the defense response of tobacco in a manner similar to an antigen, upregulating the expression of several plant defense genes [121,125]. *Tex1*, a NRPS gene, is a well-known biocontrol agent and inducer of plant defense responses. When *tex1* was disrupted in *Trichoderma virens* strain Gv29-8, 18-residue peptaibols were not formed, causing a reduction in the plant systemic resistance response against the leaf pathogen. Adding two 18-residue peptaibols to cucumber seedlings through the transpiration stream promoted the systemic response. Thus, these 18-residue peptaibols appear to play an irreplaceable role in the interactions between *Trichoderma* and plants [114].

A novel mycotoxin named acrebol, consisting of two closely related peptaibols, was reported to inhibit the respiratory chain and cause ATP depletion by activation of the oligomycin-sensitive F0F1-ATPase [60]. Trichopolyn VI isolated from the fungus *Trichoderma brevicompactum* may inhibit the mitochondrial respiratory system in *Saccharomyces cerevisiae* [106].

The trilongins form voltage-dependent, Na^+^/K^+^ permeable channels in biomembranes. Different kinds of peptaibols have various permeability ratios for Na^+^ and K^+^ ions. Trilongins with 20 and 11 residues in length isolated from the fungus *Trichoderma longibrachiatum* functioned synergistically in forming Na^+^/K^+^ permeable channels and adversely affected towards mammalian cells [71].

### 3.5 Mechanisms of antimicrobial peptide activity

#### 3.5.1 Transmembrane pore-forming mechanisms

When peptaibols contact a membrane structure, the peptide/lipid ratio is high, and the peptide molecules are vertically oriented and inserted into the bilayer to form transmembrane pores. The peptide/lipid ratio changes along with the composition of the peptide and the target lipid. Different models have been introduced to explain membrane permeability [16].

In the barrel wall model, the peptide helices form a bundle with a central lumen in the membrane, which is similar to a barrel with helical peptides serving as the staves [33,122]. The mold of transmembrane pore is unique that it is induced by alamethicin. Alamethicin attaches to, aggregates on, and inserts into directional bilayers, adopting an *α*-helical configuration. Alamethicin is hydrated with water vapor. The hydrophobic regions of peptides arrange with the core region of the lipid bilayer, while the hydrophilic regions of the peptides constitute the inner region of the pore. In the carpet model, peptides accumulate on the surface of the lipid bilayer [80]. The model indicates the activity mechanism of antimicrobial peptides like ovispirin [94], which is parallel to the membrane surface (‘in-plane’) [7]. In a carpet-like manner, peptides are electrostatically attracted to anionic phospholipid head groups at multiple locations, and cover the membrane surface. In the toroidal-pore model, peptides are spirally inserted into the membrane, causing continuous bending of the lipid monolayer to pass through the pore. As a result, the water core is surrounded by lipid head groups and inserted peptides [66]. The formation of toroidal pores is found to be similar to the pores formed by magainin, melittin and protegrins [41,66,122].

Despite membrane damage seeming to occur in different ways, the mechanisms are likely related; it has been suggested that transmembrane pores, extensive membrane rupture, and ion channels are not three totally different mechanism of action, but rather they represent a series of successive degradations [25].

#### 3.5.2 Models of intracellular killing

Antimicrobial peptides function through membrane-associated and non-membrane-associated modes of action. Generally, non-membrane modes of action can be classified as intracellular or extracellular; mechanisms include inhibiting cell wall synthesis, inhibiting DNA or protein synthesis, combining to nucleic acids, inducing autolysin or phospholipase, and inhibiting the activity of relevant enzymes [16].

Sarcotoxin IIA inhibits cell wall synthesis and shows inhibitory activity on growing bacteria [3]. Nisin (produced by *Streptococcus lactis*) and Pep 5 (produced by *Staphylococcus epidermidis*) induced autolysin production in the *Staphylococcus* cell wall [11]. The antibacterial peptide pyrrhocoricin can inhibit the ATPase action of DnaK and prevent chaperone-assisted protein folding [56]. PR-39 inhibits the synthesis of DNA and protein [14]. Buforin Ⅱ can bind to bacterial RNA and DNA, inhibiting nucleic acid function [113].

#### 3.5.3 Structural dynamics

To better understand the mechanisms of action of peptaibols, three-dimensional structures and folding dynamics are usually determined to complement in-depth bioactivity studies [5,23,104]. The relationship between peptaibol sequences and their three-dimensional structures can be determined using molecular dynamics (MD) simulations and computational modeling [[12], [111], [112]]. Amicillin, alamethicin, and zervamicin [[64], [111]] are the peptaibols that have been studied in the most detail at present. Alamethicin is known to have a high affinity for lipid bilayers; it therefore binds to the surface of lipid bilayers and can then be inserted into the membrane. Nagao et al. [73] studied the orientation of alamethicin in phospholipid bilayers using solid-state NMR in combination with molecular modeling and MD simulations[69]. The results showed that the helical axes of both N- and C-termini bent towards the central kink of the molecule with tilt angles, which facilitates insertion into the phospholipid bilayer. Hall et al. [42] made similar observations for transmembrane proteins and found that 44% of the transmembrane helices studied contained significant kinks, 35% of which were caused by proline. These results indicated that the observed bending plays a key functional role in membrane disruption or ion-channel formation.

## 4 Biosynthesis of peptaibols

### 4.1 NRPS assembly lines from different strains

The first biosynthetic research on alamethicin, a 20-residue peptaibol, in a cell-free system indicated that structures in this family are derived from the NRPS pathway. Like other peptides produced by the NRPS pathway, peptaibols skeletons are generally assembled by mega-enzymes in which multiple modules perform different functions. Each module consists of repeating adenylation (A), thiolation (T), and condensation (C) steps, which catalyze activation and selection of amino precursors, and the formation of amide bonds; formation of a TE domain promotes release of the peptide chain from the mega-enzyme [31,109,120]. The amino acid or peptide backbone can also be modified by tailoring enzymes, such as epimerization, transmethylase, or oxidoreductase, promoting structural diversity.

At present, more than 32 genera have been discovered to produce peptaibols; *Trichoderma* spp. are the main producers[68]*.* Acylation of the N-terminal, reduction of C-terminal amino acids, biogenesis and incorporation of non-proteingenic amino acids, and modular skipping in the assembly lines are of great interest to researchers in understanding peptaibol production.

Wiest et al. [120] identified *tex1*, a large NRPS gene (∼62.8 kb) with 18 modules, in *T. virens* GV29-8 via gene disruption. This was the first NRPS enzyme demonstrated to be responsible for production of peptaibols. The enzyme begins with a polyketide synthase (PKS) module, which consists of one ketosynthase (KS), one acyltransferase (AT), and one acy carrier protein (ACP). The PKS module was inferred to be responsible for acylation of the Aib residue in the N-terminal, whereas the NAD(P)H-dependent reductase (R) domain was postulated to catalyze a four-electron reduction to release the newly formed linear peptide with a hydroxyl group instead of a carboxyl in the C-terminal. Wei et al. [119] subsequently showed that *tex1* can only produce the 18-mer peptaibols in *T. virens* GV29-8, whereas 14- and 11-mer peptaibols were synthesized by a 14-residue NRPS gene, *tex2* (∼50.1 kb) [119]. The difference between the 11- and 14-mer peptaibols is a lack of amino acids at position 4, 5, and 6 in the 11-mer, which means there is an alternative skipping of modules in the NRPS biosynthesis pathway. Researchers have proposed two skipping mechanisms: one is the direct intermediate transfer between nonadjacent modules, and the other involves nonfunctional T or C domains in biosynthesis. Module skipping is a common phenomenon during biosynthesis of many types of non-ribosomal peptides, while it was the first demonstrated in peptaibol synthesis.

A gene cluster encoding a 20-aa peptaibol was identified in *T. longibrachiatum* (Tl). This cluster contains the NRPS gene SMF2FGGW_105489 (69.5 kb), which is the longest peptaibol NRPS gene reported to date. Komon-Zelazowska et al. [54] characterized a 19-module peptaibol synthetase gene, *pbs1* (66.3 kb) from the genome of *Hypocrea atroviridis.* This gene allowed production of both 19- and 20-aa peptaibols: atroviridin and PBS1. Atroviridin contains a triple amino acid sequence (Aib5-Aib6-Gln7), whereas PBS1 possesses only one Aib residue preceding the Gln residue, which means that the Aib module in the NRPS may be used twice. A similar mechanism has also been reported in other fungal NRPSs, but it has not been elucidated for peptaibols. Du et al. [32] identified a 21-module NRPS cluster, NP_T2_, from *Tolypocladium sp.* Sup5-1 (T2) [32]. Interestingly, the NRPS gene was split into two parts. The N-terminal fragment contained KS and AT domains followed by eight modules of NRPS domains (30 kb), and the C-terminal fragment contained 13 modules of NRPS domains (42 kb). Further studies have supported that the NP_T2_ cluster likely synthesizes the 21- and 22-aa peptaibols gichigamins A–G.

### 4.2 Biosynthesis of non-proteingenic α, α-dialkyl amino acids

Peptaibols skeletons are rich in non-proteingenic amino acids such as Aib and Iva. The presence of these unusual amino acids may enhance helical structures, minimize protein degradation, and improve bioactivity. However, the biogenesis of these amino precursors has long remained a mystery. Researchers presumed that Aib could be formed from methylation of L-alanine catalyzed by methyltransferase using adenosyl-methionine as a methyl donor [61]. However, methylation on an electrophilic α-C is likely a difficult task. For this reason, some researchers believed these structures did not really occur in the biosphere but had extraterrestrial origins [17]. Recently, Ikuro Abe discovered via gene disruption experiments that two enzymes (TqaM and TqaL from *E. salmosynnemata*) were responsible for the formation of Aib, although the catalyzing functions of these two genes have not been directly verified [18]. Raap et al. [83] added ^15^N _DL_-Iva to the culture medium of *E. salmosynnemata* and detected production of 15-aa peptaibols with both ^15^N-isotoped D-Iva and Aib residues. This indicated that Iva could be metabolized and used for the biosynthesis of Aib [83]. However, more conclusive evidences are still required to fully confirm the biogenesis mechanisms of these unusual amino acids.

### 4.3 Regulation of peptaibol biosynthesis

To improve peptaibol biosynthesis (i.e., to produce higher levels and more diversified structures), researchers have made adjustments at the macro level (e.g., altering fermentation culture conditions) and at the molecular levels (e.g., manipulating regulatory factor). It has been shown that many peptaibols are preferentially produced on the surface of cultures, with only a few cases of peptaibols being produced in submerged cultures. For example, *Trichoderma arundinaceum* strain 63 C-1 produces two micro-heterogeneous groups of peptaibols (suzukacillins) under submerged culture conditions but with very low yield. In that situation, peptaibol production can be promoted by adding an insoluble component (such as cellulose) to the submerged cultures [15]. Schirmbock et al. [96] found that peptaibols were only produced by *T.harzianum* in liquid minimal medium with the presence of cell walls, suggesting that cell-wall-degrading enzymes could regulate peptaibol formation.

Supplementation of specific amino acids in culture medium can also influence the formation of peptaibols. The addition of Aib to a culture of *T. harzianum* M-902608, the producer of 18-aa trichorzins PA and 14-aa harzianins PC, led to the discovery of a series of new peptaibols, in which all Iva residues were replaced by Aib residues [62]. The addition of Glu into *T. longibrachiatum* M-853431 culture was reported promote synthesis of longibrachins synthesis [62]. When free Aib was added to *E. salmosynnemata*, the producer of the 15-aa peptaibols Zrv-IIA and Zrv-IIB, the ratio of Zrv-IIA: Zrv-IIB greatly increased; in contrast, with the addition of _DL_-Iva, only Zrv-IIB was produced [83].

Peptaibol biosynthesis is also influenced by some regulatory factors. In *H. atroviridi*, Monika Komon-Zelazowska et al. [54] verified that atroviridins were not formed during the vegetative growth phase. However, when the colonies began sporulation, atroviridins started accumulating in the culture. They further found with disruption experiments that biosynthesis of atroviridin was affected by two blue light regulators, BLR1 and BLR2, together with the negative sporulation regulator G*_α_*-protein GNA3 [54].

In 2018, Gómezrodríguez et al. found that deletion of the *tgf-1* gene, which encodes a histone acetyltransferase, greatly hindered transcription of the NRPS gene *pbs-1* during the first 48 hour of *H. atroviride* cultivation [123]. Alberto Alonso Monroy et al. [72] reported an indirect influence on production of paracelsin caused by a CRE1-regulated cluster that was also responsible for light-dependent production of dihydrotrichotetronin. Yu-Zhong Zhang et al. (2019) later found that disruption of *Tlstp1*, a conserved sugar transporter in the genome of *T. longibrachiatum*, significantly impaired growth and conidiation, but markedly enhanced peptaibol production. Further transcriptome analyses indicated that *tlx1* and *tlx2*, which are involved in peptaibol biosynthesis, were upregulated in the transcriptome of *tlstp1* mutants [126]. This indicated that TlSTP1 is an important regulating factor affecting vegetative growth and peptaibol production. Phylogenetic analyses showed that homologues of TlSTP1 with high sequence identities (87–96%) are distributed among many peptaibol-producing *Trichoderma* species.

Despite these reports, the regulatory network controlling peptaibol synthesis is still largely unknown. More in-depth studies should be undertaken to elucidate peptaibol metabolism, *e.g.*, the metabolic flux of amino acid precursors, for which peptaibols compete with other NRPS pathways. This would promote understanding of their elaborate biosynthetic machinery and facilitate metabolic engineering efforts to improve peptaibol yield.

## 5 Conclusion and outlook

To date, more than 1000 peptaibols have been discovered in fungi. Despite reports of a wide variety of biological activities, most peptaibols have only been assessed for their antimicrobial properties. Peptaibols commonly exert antimicrobial effects through membrane permeabilization. This may ineluctably be associated with toxicity to mammalian cells when applying these compounds in human disease treatments due to the similar membrane structures. In light of this, computational chemistry and computer aided drug design (CADD) may aid in producing groups of refined structures based on structure–activity relationships; combined with solid-phase synthesis, it will become more efficient to generate targeted peptaibol entities.

Synthetic biology and combinatorial techniques are advanced tools for producing diversified structures. However, the oligomeric amino acid skeleton of a peptaibol is usually encoded by one intact NRPS mega-enzyme containing more than 10 modules, over 40 kb in size and spanning an entire chromosome. This makes it challenging to reconstruct and express in a heterologous host. Tools for direct cloning of large DNA fragments based on target-directed genome mining (TAR), Cas9-Gibson, and *Escherichia coli λ* Red gene recombination (RED/ET) techniques may provide solutions to feasibly capture large NRPS genes for bioengineering of peptaibols [1,37,46,50,55].

Finally, the development and application of spectroscopic and biochemical tools in natural product discovery research have greatly facilitate the isolation and characterization of diversified peptaibols with promising bioactivity. With increasing efforts devoted into pharmacological and pharmaceutical studies, peptaibols are expected to be developed into human disease therapies within the foreseeable future.

## Declaration of Competing Interest

The authors declare that they have no known competing financial interests or personal relationships that could have appeared to influence the work reported in this paper.
